# A multi-modality and multi-granularity collaborative learning framework for identifying spatial domains and spatially variable genes

**DOI:** 10.1093/bioinformatics/btae607

**Published:** 2024-10-17

**Authors:** Xiao Liang, Pei Liu, Li Xue, Baiyun Chen, Wei Liu, Wanwan Shi, Yongwang Wang, Xiangtao Chen, Jiawei Luo

**Affiliations:** College of Computer Science and Electronic Engineering, Hunan University, Changsha 410082, China; College of Computer Science and Electronic Engineering, Hunan University, Changsha 410082, China; College of Computer Science and Electronic Engineering, Hunan University, Changsha 410082, China; Computer Science, Tuskegee University, State of Alabama 36088, United States; College of Computer Science and Electronic Engineering, Hunan University, Changsha 410082, China; College of Computer Science and Electronic Engineering, Hunan University, Changsha 410082, China; College of Computer Science and Electronic Engineering, Hunan University, Changsha 410082, China; College of Computer Science and Electronic Engineering, Hunan University, Changsha 410082, China; College of Computer Science and Electronic Engineering, Hunan University, Changsha 410082, China

## Abstract

**Motivation:**

Recent advances in spatial transcriptomics technologies have provided multi-modality data integrating gene expression, spatial context, and histological images. Accurately identifying spatial domains and spatially variable genes is crucial for understanding tissue structures and biological functions. However, effectively combining multi-modality data to identify spatial domains and determining SVGs closely related to these spatial domains remains a challenge.

**Results:**

In this study, we propose spatial transcriptomics multi-modality and multi-granularity collaborative learning (spaMMCL). For detecting spatial domains, spaMMCL mitigates the adverse effects of modality bias by masking portions of gene expression data, integrates gene and image features using a shared graph convolutional network, and employs graph self-supervised learning to deal with noise from feature fusion. Simultaneously, based on the identified spatial domains, spaMMCL integrates various strategies to detect potential SVGs at different granularities, enhancing their reliability and biological significance. Experimental results demonstrate that spaMMCL substantially improves the identification of spatial domains and SVGs.

**Availability and implementation:**

The code and data of spaMMCL are available on Github: Https://github.com/liangxiao-cs/spaMMCL.

## Introduction

Spatial transcriptomics (STs) technologies can measure gene expression and spatial information within tissues (Moses and Pachter 2022). With the development of ST technologies such as 10× Visium (Hudson and Sudmeier 2022), Slide-seq (Rodriques *et al.* 2019, Stickels *et al.* 2021), and Stereo-seq (Chen *et al.* 2022), numerous ST data have been constructed. The complexity of ST data poses great challenges to related tasks, such as identifying spatial domains (Wang *et al.* 2023a) and discovering spatially variable genes (SVGs) (Zhang *et al.* 2023).

In the field of ST research, the identification of spatial domains (i.e., regions exhibiting similar spatial expression patterns) is a crucial area of study (Rahman *et al.* 2023). It allows for a comprehensive understanding of the spatial location and function of cells or spots. Recently, deep learning-based computational methods have been extensively applied in the recognition of spatial domains. For example, SEDR integrates a deep autoencoder with a masked self-supervised learning framework and a variational graph autoencoder to formulate representations of gene expression (Xu *et al.* 2024). STAGATE employs a graph attention auto-encoder to learn representations that reflect the spatial proximity and gene expression similarities (Dong and Zhang 2022). MuCoST constructs both a co-expression graph and a shuffled graph, using a contrast loss function to extract consistent representations from both graphs (Zhang *et al.* 2024). GraphST employs an iterative aggregation mechanism of graph neural networks to synthesize gene expression from neighboring points (Long *et al.* 2023). CCST utilizes the Deep Graph Infomax (Velickovic *et al.* 2019) framework to learn representations, focusing on maximizing mutual information between local and global graph features (Li *et al.* 2022). However, these methods are susceptible to noise interference due to their sole reliance on gene expression and spatial information. Given the aforementioned limitations, there has been a continual emergence of methods including histological images as Supplementary Data. For instance, stSME integrates spatial locations, histological images, and gene expression to adjust gene expression values (Pham *et al.* 2023). ConGI, through a contrast learning strategy, aligns gene expression and histological images in a low-dimensional space to learn a common representation (Zeng *et al.* 2023). While these methods that leverage multi-modality ST data can identify different domains, they suffer from a phenomenon known as modality bias, where different modalities contribute inconsistently to the results (Zang *et al.* 2024). One of the most common issues is that these methods might overlook valuable information present in the weaker modalities.

Concurrently, extensive computational models for identifying SVGs, i.e., genes with significantly variable expression across different spatial locations. This has promoted a deeper understanding of gene expression patterns between different tissue regions. For example, SpatialDE quantifies the spatial variability of gene expression through a Gaussian Process model (Svensson *et al.* 2018). SINFONIA identifies SVGs using an integrated strategy that combines global and local spatial autocorrelation (Jiang *et al.* 2023). SpaGCN detects SVGs enriched in each spatial domain through domain-guided differential expression analysis (Hu *et al.* 2021). BSP defines a large and small neighborhood for each spot, calculates the ratio of variances of the local mean gene expression within these neighborhoods for identifying SVGs (Wang *et al.* 2023b). However, there is a limitation in detecting SVGs only by analyzing differential expression between certain regions, which makes it impossible to capture gene expression changes at different spatial scales.

In view of the abovementioned limitations, we propose a two-stage framework named spaMMCL to identify spatial domains and detect SVGs. Specifically, spaMMCL consists of the multi-modality learning module (MML) for spatial domains identification and multi-granularity learning module (MGL) for SVGs detection. In MML module, we first introduce a feature mask-like method to randomly mask a certain proportion of gene expression, mitigating the adverse effects of modality bias. We then design a shared graph autoencoder to jointly learn and fuse gene and image features. Finally, we employ graph self-supervised learning, addressing noise that arises after the fusion of gene and image features. With these refined spot representations, we apply a clustering method to effectively partition the spatial domains. In MGL module, to enhance the reliability and biological significance of SVGs identification, we integrate various strategies to comprehensively detect potential SVGs at different granularities. Granularity denotes the hierarchical level at which a system is composed of distinguishable components (Hobbs 1990). Extensive experiments are performed on different datasets, and the results demonstrate that spaMMCL has significant advantages in spatial domain and spatially variable gene identification tasks.

## Materials and methods

### Overview of spaMMCL

**Figure 1.**
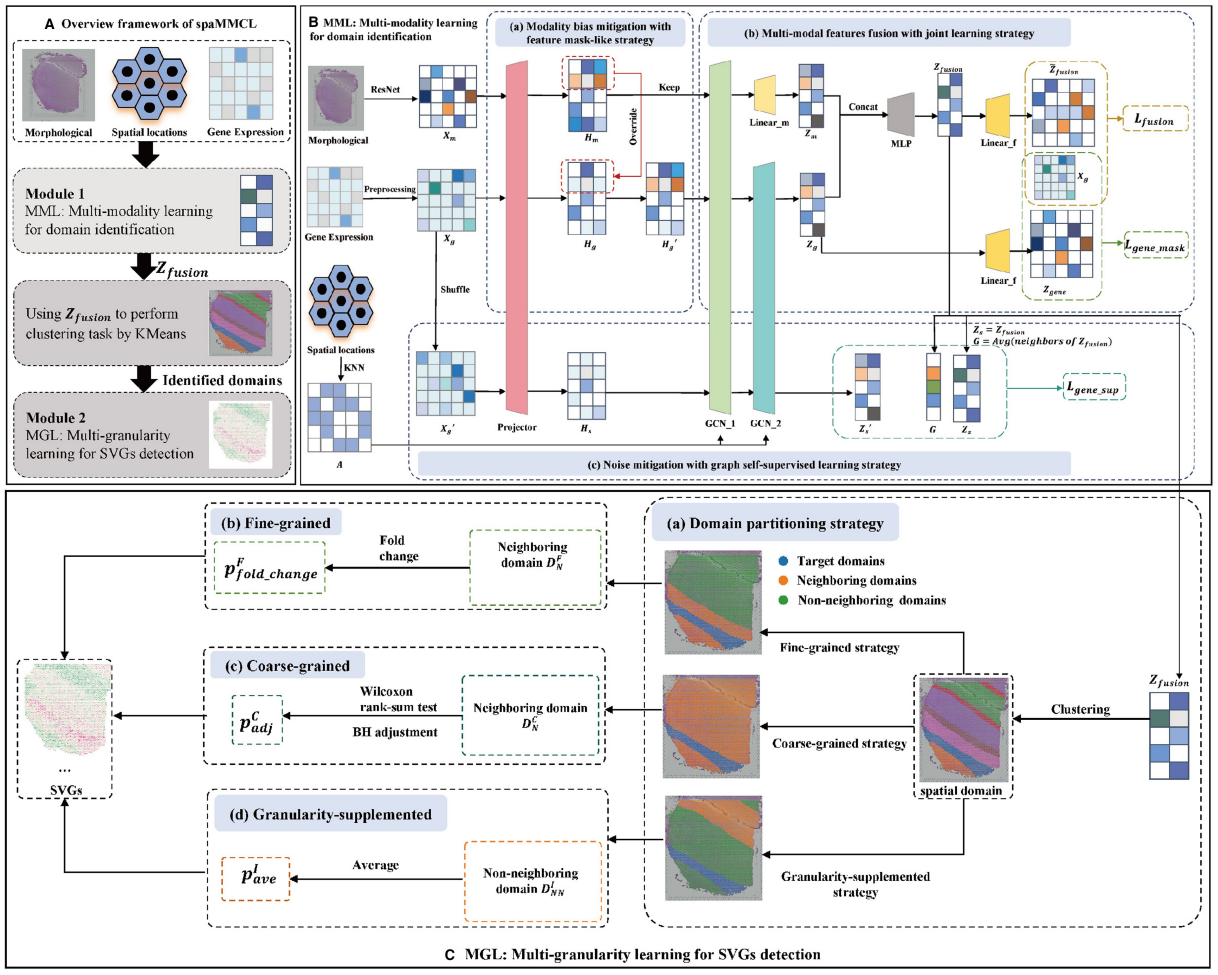
The overall framework of spaMMCL. (A) Workflow of spaMMCL. spaMMCL uses multi-modal data as input. The framework comprises two modules: MML and MGL. (B) MML module. The MML module is designed to learn from multi-modal data. It includes three components: (a) modality bias mitigation with feature mask-like strategy, (b) multi-modal features fusion with joint learning, and (c) noise mitigation with graph self-supervised learning. (C) MGL module. The MGL module employs a granularity-guided approach. It includes four components: (a) domain partitioning strategy, (b) fine-grained screening strategy, (c) coarse-grained screening strategy, and (d) granularity-supplemented constraint strategy.

In this section, we propose a novel method named spaMMCL. As shown in Fig. 1A, the spaMMCL framework consists of two components: multi-modality learning module (MML) for spatial domains identification and multi-granularity learning module (MGL) for SVGs detection. The MML module contains three components: modality bias mitigation with feature mask-like strategy, multi-modal features fusion with joint learning and noise mitigation with graph self-supervised learning (Fig. 1B). The MGL module also contains three components: a fine-grained screening strategy, a coarse-grained screening strategy, and a granularity-supplemented constraint strategy (Fig. 1C). spaMMCL first employs MML module to collaboratively learn gene expression, histological images and spatial context, while accounting for modal deviation phenomena during the integration of multi-modal data. Subsequently, spaMMCL employs MGL module, a granularity-guided approach, to identify more accurate spatial domain-specific SVGs at different scales.

### Datasets and preprocessing

ST data are composed of three essential modalities: gene expression counts, histological images, and spatial coordinates. For gene expression counts, we selected the top 3000 highly variable genes to construct the initial gene expression feature embedding $X_{g}\in R^{n\times g}$ with *n* spots and *g* genes. For histology images, given the original patches cropped from histology images at each spot, we applied ResNet-152 (He *et al.* 2016) to obtain the initial image feature embedding $X_{m}\in R^{n\times m}$ with *n* spots and *m* features dimensions. To evaluate the effectiveness of spaMMCL, we collected four ST datasets: human dorsolateral prefrontal cortex (DLPFC) with 12 slices, human breast cancer (HBC), and mouse brain, all from 10× Visium platform (Hudson and Sudmeier 2022), and mouse olfactory bulb from Stereo-seq platform (Chen *et al.* 2022). A summary of these datasets is provided in Supplementary Table S2.

### MML: multi-modality learning for domain identification

#### Modality bias mitigation with feature mask-like strategy

Given the initial gene expression features $X_{g}\in R^{n\times g}$ and image features $X_{m}\in R^{n\times m}$, we convert them to the same dimension using two linear layers Linear(·), respectively, and apply a projection layer PG(·) consisting of a one-layer neural network to map them into a shared space. Formally, the post-projection embedding $H_{g}\in R^{n\times d_{1}}$ and $H_{m}\in R^{n\times d_{1}}$ are obtained. Then, we randomly sample a subset $SS_{\mathrm{sub}}$ from the entire spots set *SS*, and replace each gene expression value in the subset $SS_{\mathrm{sub}}$ with corresponding image value. Formally, the new gene expression matrix $H_{g}^{\prime }$ is defined as follows:

(1) $$\begin{array}{c} H_{g}^{\prime }=\{\begin{array}{c} H_{m},\mathrm{spot}\in SS_{\mathrm{sub}}\\ H_{g},\mathrm{spot}\notin SS_{\mathrm{sub}} \end{array} \end{array}$$

#### Multi-modal features fusion with joint learning strategy

After obtaining gene features $H_{g}^{\prime }$ and image features $H_{m}$, we jointly learn them using shared graph convolutional networks (GCNs). Thus, we calculate the Euclidean distances between spots using the spatial coordinates and use the *k*-nearest neighbors to construct a spot-to-spot relational graph. Formally, we utilize an adjacency matrix ***A*** to represent this graph, where $A_{ij}=1$ if *i* and *j* are neighbors, and 0 otherwise. To learn more informative and common embeddings, we apply a shared GCNs framework based on *A* to simultaneously extract the graph structure and spot characteristics. In particular, for *k*th modality embedding $H_{k}$ (k∈{g,m}), we propose a mapping function $f(A,H_{k},\theta )\to Z_{1}^{\left(k\right)}$ to map the gene features and image features into a latent space, respectively, where θ represents the shared model parameters. Following Kipf (Kipf and Welling 2016), the final embedding of *k*th modality is $Z_{1}^{\left(k\right)}\in R^{n\times d_{2}}$.

Moreover, to further balance individual and common features of multi-modal data, we apply a graph neural network GCN(·) and a one-layer linear layer $\mathrm{Linea}r_{m}(\cdot )$ to obtain the gene representations $Z_{g}\in R^{n\times d_{3}}$ and the image representations $Z_{m}\in R^{n\times d_{4}}$, respectively. Then, we utilize an MLP(·) and a concatenation layer Concat(·) to fuse the information from $Z_{g}$ and $Z_{m}$ to obtain the final representation $Z_{\mathrm{fusion}}\in R^{n\times d_{3}}$. To fully capture the commonalities and specificities of multi-modal features, we design two reconstruction loss functions for joint learning. As described below:

(2) $$Z_{\mathrm{fusion}}=\mathrm{MLP}(\mathrm{Concat}(Z_{g},Z_{m}))$$

(3) $$L_{\mathrm{fusion}}=\sum_{i=1}^{SS}{\left\|X_{g}-{\tilde{Z}}_{\mathrm{fusion}}\right\|}_{F}^{2}$$

(4) $$L_{\mathrm{gene}\_\mathrm{mask}}=\sum_{i=1}^{SS_{\mathrm{sub}}}{\left\|X_{g}-Z_{\mathrm{gene}}\right\|}_{F}^{2}$$

where $Z_{\mathrm{gene}}=\mathrm{Linea}r_{f}(Z_{g})\in R^{n\times g}$ and ${\tilde{Z}}_{\mathrm{fusion}}=\mathrm{Linea}r_{f}(Z_{\mathrm{fusion}})\in R^{n\times g}$. $\mathrm{Linea}r_{f}$ are a one-layer linear layer to convert gene features into the original space. $L_{\mathrm{fusion}}$ is to learn multi-modal features simultaneously, and $L_{\mathrm{gene}\_\mathrm{mask}}$ is to prevent the loss of gene expression information.

#### Noise mitigation with graph self-supervised learning strategy

To alleviate the noise problem after fusing gene and image features, we introduce a graph self-supervised learning framework to make representations less noisy. This part consists of three key steps:

- Constructing corrupted graph. Given the initial gene expression $X_{g}$, we create a shuffled features $X_{g}^{\prime }$ by randomly shuffling the gene expression vectors among the spots.
- Encoding spots features. Using the shuffled gene features $X_{g}^{\prime }$, we use a GNN-based encoder to generate the corrupted embedding of spots $Z_{s}^{\prime }\in R^{n\times d_{3}}$. Note that the real embedding of spots is $Z_{s}=Z_{\mathrm{fusion}}\in R^{n\times d_{3}}$.
- Designing positive/negative pairs. Inspired by GraphST, the local context $G_{i}$ of spot *i* is defined as a sigmoid function applied to the mean of the representations of its direct neighbors (Long *et al.* 2023), unlike DGI (Velickovic *et al.* 2019). Thus, given the original representations $Z_{s}$, the corrupted representations $Z_{s}^{\prime }$ and local context *G*, we can form a positive pair $<Z_{i}^{\left(s\right)},G_{i}^{\left(s\right)}>$ and a negative pair $<Z_{i}^{\prime (s)},G_{i}^{\left(s\right)}>$ for a spot *i*.

Formally, we use binary cross-entropy (BCE) to construct the supervised contrastive learning (SCL) (Khosla *et al.* 2020) loss for spots:

(5) $$\begin{array}{c} \begin{array}{c} L_{\mathrm{gene}\_\sup}=-\frac{1}{2n}(\sum_{i=1}^{n}(E_{\left(X,A\right)}[\text{log}\;\Phi (Z_{i}^{\left(s\right)},G_{i}^{\left(s\right)})]\\ +E_{\left(X^{\prime },A\right)}[\text{log}(1-\Phi (Z_{i}^{\prime (s)},G_{i}^{\left(s\right)}))])) \end{array} \end{array}$$

and the total loss of MML module can be expressed as follows:

(6) $$L=\alpha L_{\mathrm{gene}\_\mathrm{mask}}+L_{\mathrm{fusion}}+L_{\mathrm{gene}\_\sup}$$

For all datasets, α =10. Finally, to identify spatial domains, we use the representation $Z_{\mathrm{fusion}}$ to perform clustering task. The parameter settings are detailed in Supplementary Note S3.

### MGL: multi-granularity learning for SVGs detection

#### Fine-grained screening strategy

Given a specific target domain *D*, the SVGs detection process is as follows:

- Constructing neighboring spatial domain. We adopt the spaGCN (Hu *et al.* 2021) framework to identify a small number of neighbors. Specifically, we draw a circle with a predefined radius around each spot in the target domain, considering as neighbors any spots from non-target domains that fall within this circle. This configuration results in each spot having approximately 10 neighbors. The neighbors of all spots collectively form a neighboring set. For each non-target domain, a domain is classified as a neighboring domain if more than 50% of its spots are found within the neighboring set. Subsequently, all neighboring domains are aggregated to construct a neighboring domain $D_{N}^{F}$.
- Quantifing differentially expressed genes. The fold change of genes between target domain *D* and neighboring domain $D_{N}^{F}$ is calculated as follows:
  (7) $$p_{fold\_change}^{F}=\frac{{\tilde{x}}_{D}}{{\tilde{x}}_{D_{N}^{F}}}$$
  where ${\tilde{x}}_{D}$ is the average gene expression vector for genes in the target domain *D* and ${\tilde{x}}_{D_{N}^{F}}$ is the average for the neighboring domain $D_{N}^{F}$. Genes with a fold change above 1.5 are considered SVGs of fine-grained.

#### Coarse-grained screening strategy

Given a specific target domain *D*, we exclude *D* and utilize the remaining domains to form its neighboring domains $D_{N}^{C}$. Finally, we define genes with an $p_{\mathrm{adj}}^{C}$ below 0.05 as SVGs of Coarse-grained.

#### Granularity-supplemented constraint strategy

To improve the reliability of SVGs, we introduce a granularity-supplemented constraint strategy for collaboratively screening SVGs identified at both fine and coarse granularities. Specifically, using the neighboring domains identified in fine-grained analysis, we first extract a portion of the non-neighborhood domains to form a new comparison domain. Then, we apply the same method as used in the fine-grained approach to obtain the average expression ${\tilde{x}}_{D}$ and ${\tilde{x}}_{D_{NN}^{I}}$ for genes in the target domain *D* and the comparison domain $D_{NN}^{I}$, respectively. This strategy uses the following definition:

Definition 1. *For a gene* $g_{i}$*, given the average expression value* $x_{g_{i}}$*in* ${\tilde{x}}_{D}$*and* ${\tilde{x}}_{g_{i}}$*in* ${\tilde{x}}_{D_{NN}^{I}}$*, if* $x_{g_{i}}>{\tilde{x}}_{g_{i}}$*then* $g_{i}$*is adopted as the SVGs of the target domain D, otherwise not.*

In the end, MGL module adopts the intersection of SVGs identified by these three strategies as the final set of SVGs for each domain. It constructs a more accurate and comprehensive analysis framework for SVG identification by analyzing gene expression at different granularities.

## Results

### Benchmarking spaMMCL

We first used the human dorsolateral prefrontal cortex (DLPFC) dataset to assess the performance of spaMMCL. We evaluated clustering performance with the ARI (Supplementary Note S1) and assessed the spatial autocorrelation of SVGs with the Moran’s I (Supplementary Note S2).

**Figure 2.**
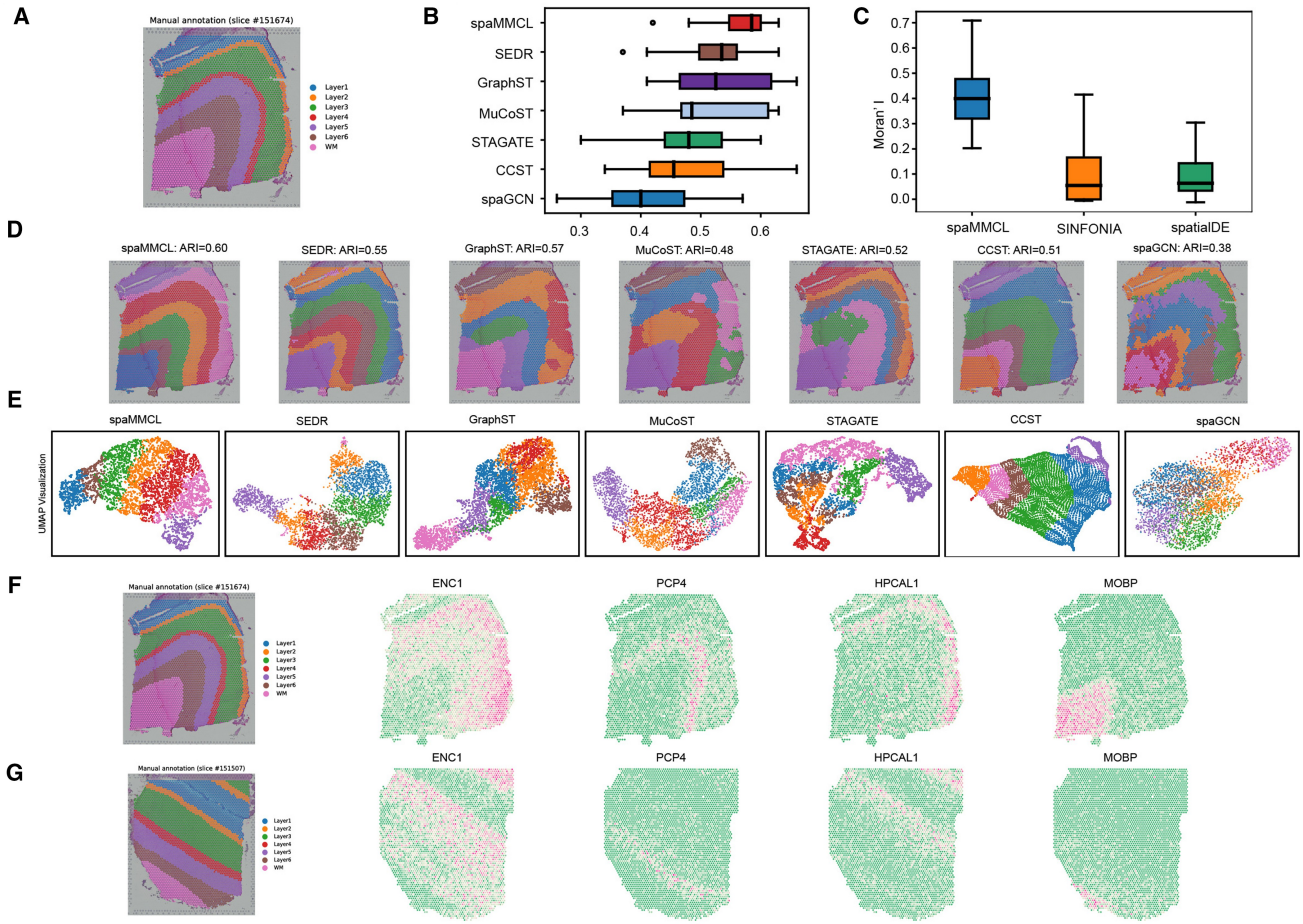
Benchmarking spaMMCL. (A) Manual annotated of slice 151674. (B) Boxplot of ARI for spaMMCL and comparison methods across 12 slices. (C) Boxplot of Moran’s I for spaMMCL and comparison methods of 151674. (D) Domain identification of 151674 by all methods. (E) UMAP visualization of 151674. (F), (G) Spatial expression patterns of SVGs detected by spaMMCL of 151674, and the transfer of these SVGs to a different slice 151507.

We assessed overall performance across the 12 slices of DLPFC. spaMMCL consistently outperformed other methods, achieving a median ARI value of 0.585 and a mean ARI value of 0.563 (Fig. 2B). Next, we evaluated spatial domain identification using slice 151674 (Fig. 2A). Figure 2D shows that spaGCN exhibited limited performance, identifying only Layer 1 and Layer 2 without delineating clear inter-layer boundaries. Domains identified by CCST closely matched the manually labeled layers, but it entirely missed Layer 4. GraphST could effectively delineate the hierarchy of slices, but it could not accurately identify the Layer 2 and Layer 4 layers with few spots, which led to suboptimal visualizations. UMAP visualization further demonstrated the effectiveness of our method (Fig. 2E).

Based on the spatial domains obtained, we used our multi-granularity approach to detect SVGs and compared them with those identified by SINFONIA and SpatialDE. In particular, the median Moran’s I value of our model achieved 0.41 (Fig. 2C). This difference is primarily due to SINFONIA and SpatialDE not accounting for spatial domains. Furthermore, the SVGs could be effectively transferred to slice 151507 (Fig. 2F and G). Although Layer 3 was divided into two parts in slice 151507, due to the accuracy of spatial domain identification, we still detected ENC1 as its SVG. Previous studies have indicated that ENC1 may play a protective role in the brain’s response to neuropathological damage and aid in maintaining cognitive functions (White *et al.* 2017). These findings further highlighted the superior performance of spaMMCL in detecting SVGs.

### spaMMCL reveals heterogeneity in human breast cancer

**Figure 3.**
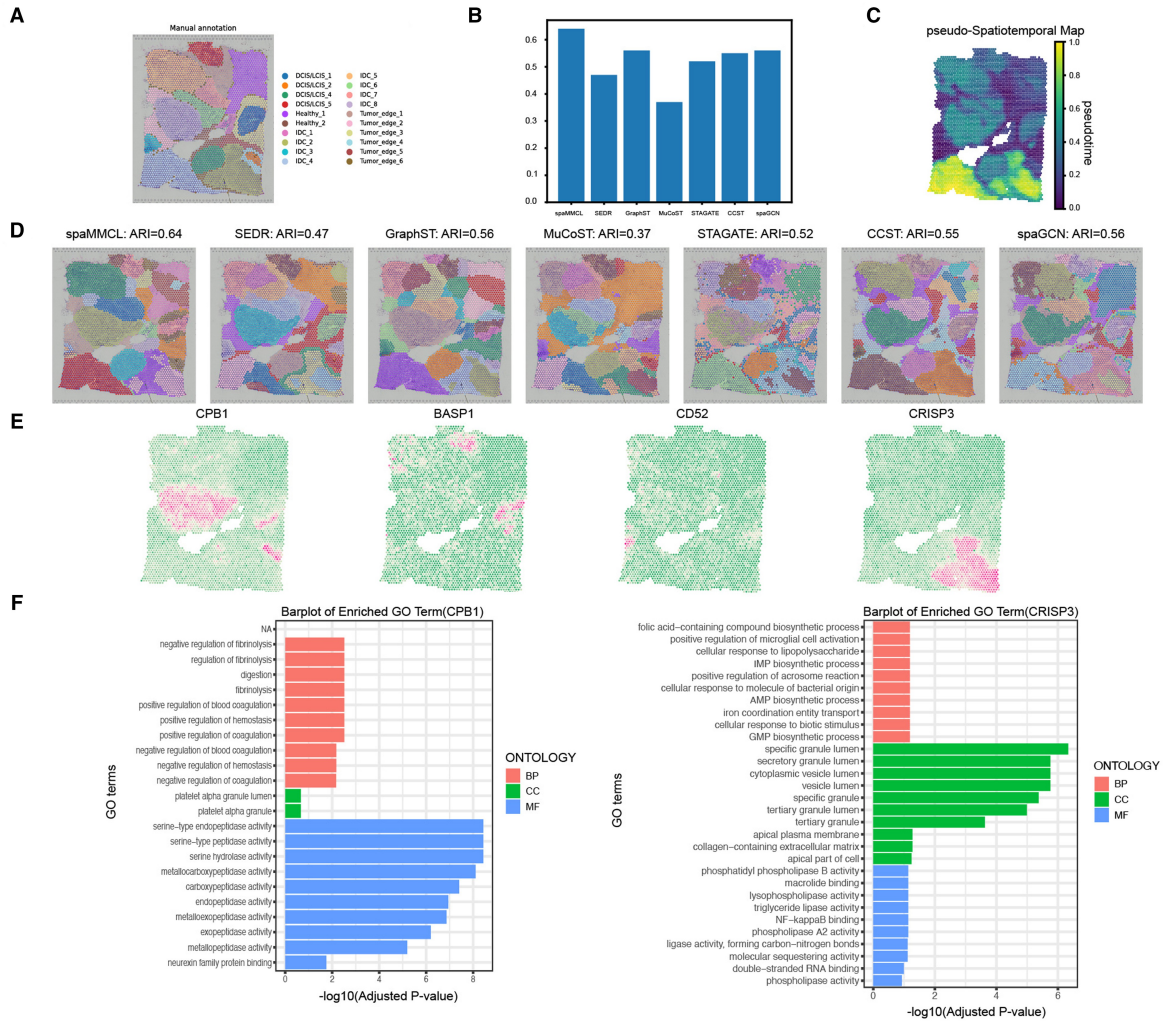
spaMMCL can dissect the heterogeneity of human breast cancer. (A) Manual annotated of HBC. (B) Barchart of ARI for spaMMCL and comparison methods. (C) pSM values. (D) Domain identification by all methods. (E) Spatial expression patterns of SVGs detected by spaMMCL. (F) GO enrichment analysis of SVGs.

Next, we analyzed the human breast cancer (HBC) dataset, including four primary morphotypes: Ductal carcinoma in situ/lobular carcinoma in situ (DCIS/LCIS), invasive ductal carcinoma (IDC), healthy tissue, and tumor edge (Fig. 3A).

Figure 3D shows that the domains identified by spaMMCL closely corresponded to the manually labeled layers. Notably, domains such as IDC_5 were accurately identified, and each domain exhibiting more coherent segmentation. MuCoST achieved the worst results, with overlapping spots between the domains. STAGATE and SpaGCN identified more distinguishable domains, but many of the identified domains lacked clear boundaries. GraphST obtained sub-optimal results due to an effective contrast learning strategy but overlooked the promoting role of histological images. In contrast, spaMMCL integrated multi-modal data through MML module, resulting in better performance. Therefore, spaMMCL attained the highest ARI value of 0.64 (Fig. 3B). A pseudo-spatiotemporal map (pSM) (Ren *et al.* 2022) was constructed using the embeddings from spaMMCL (Fig. 3C). The pSM exhibited a stratified architecture with distinct and gradual coloration, reflecting the sequential transition from malignant to non-malignant tissue states.

Guided by the identified domains, we investigated SVGs (Fig. 3E). We performed a gene ontology (GO) analysis and presented the results for CPB1 and CRISP3 (Fig. 3F). We found that both genes are closely associated with the development of cancer, playing distinct roles in different types of tumors. The expression level of CPB1 is closely related to disease progression in DCIS (Kothari *et al.* 2021). The expression of CRISP3 is intimately connected with the characteristics of cancer stem cells, playing a key role in the self-renewal and multilineage differentiation potential of tumors (Wang *et al.* 2024). These studies emphasized the significant biological importance of the SVGs that we identified.

### spaMMCL precisely identifies tissue structures in the mouse brain

**Figure 4.**
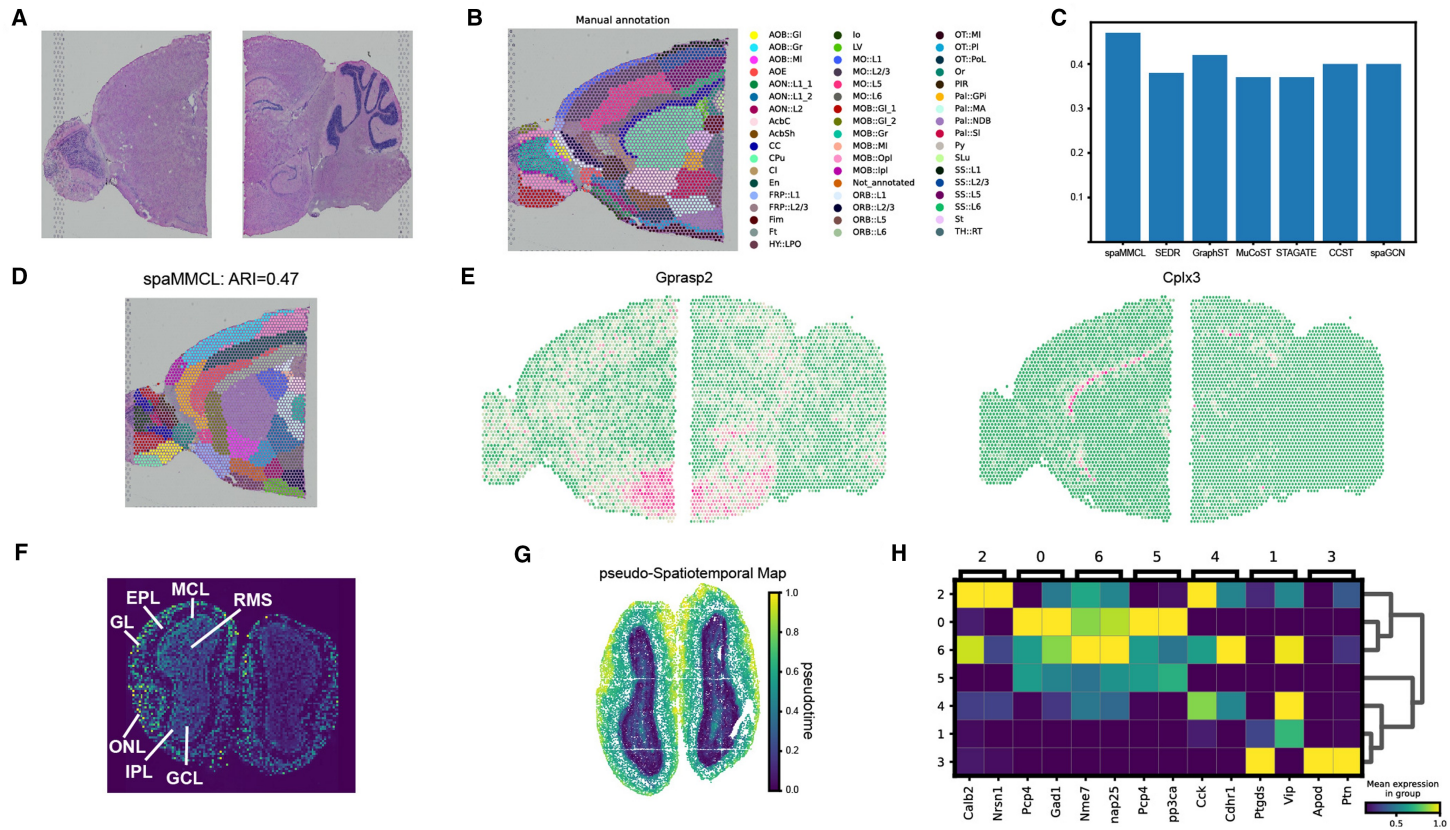
(A) Two slices of mouse brain. (B) Manual annotated for mouse anterior brain slice. (C) Barchart of ARI for spaMMCL and comparison methods of mouse anterior brain slice. (D) Domain identification by spaMMCL. (E) The spatial expression pattern of SVGs in anterior brain slice, with these SVGs also transferable to posterior brain slice. (F) Mouse olfactory bulb from Stereo-seq platform. (G) pSM values. (H) Marker genes.

Then, we harnessed the SVGs identified by spaMMCL to illustrate their application in horizontal integration. For this purpose, we selected two slices of mouse brain, including the anterior and posterior regions (Fig. 4A). We evaluated spatial domain identification using anterior brain slice (Fig. 4B). The spaMMCL achieved the highest ARI value (Fig. 4C). CCST and spaMMCL were largely congruent with the established anatomical structures. However, CCST exhibited deficiencies in discerning delicate structures (Supplementary Fig. S1). In contrast, spaMMCL was capable of distinctly delineating these areas (Fig. 4D).

Our study revealed that the SVGs detected in the anterior brain slice can be effectively transferred and applied to the posterior brain slice. Particularly, at the junction between two slices, we observed the expression patterns of specific SVGs. The SVGs exhibited a consistent and continuous distribution across the two slices (Fig. 4E). This finding confirmed the crucial role of SVGs in achieving effective horizontal integration between different slices.

### spaMMCL is suitable for spatial transcriptomics data with various platforms

After that, we validated spaMMCL using the mouse olfactory bulb dataset (Fig. 4F) derived from the Stereo-seq platform (Chen *et al.* 2022). spaMMCL demonstrated a clear pattern that closely corresponded to the annotated layers (Supplementary Fig. S2). Furthermore, pSM values were lowest in the external plexiform layer (EPL) and exhibited a gradual increase in both directions away from this layer (Fig. 4G). This pattern aligned with the established developmental sequence of these layers (Ren *et al.* 2022). Additionally, we examined the expression of corresponding marker genes for each layer (Fig. 4H). This indicated that the embeddings generated by spaMMCL accurately reflected the developmental and spatiotemporal relationships between spots.

### Ablation study

To further investigate the mechanism of spaMMCL, we conducted a series of ablation studies on the human breast cancer dataset. In these experiments, we systematically removed modal deviation mitigation (w/o-dev), multi-modal features joint learning (w/o-sha), and graph self-supervised learning (w/o-con) to assess their individual contributions for model performance. As shown in Supplementary Fig. S3, w/o-dev, w/o-sha, and w/o-con all showed much lower ARI and NMI values than spaMMCL. The results clearly demonstrated the advantages of collaborative learning in spaMMCL.

## Conclusion

This study presents an innovative method, spaMMCL, which consists of two core modules: the MML module is dedicated to identifying spatial domains, and the MGL module focuses on detecting SVGs. To validate the performance of our model, we tested spaMMCL on four real spatial transcriptomics datasets. The experimental results demonstrated that our model achieved competitive performance in spatial domain identification compared to state-of-the-art methods. Simultaneously, our method effectively detected SVGs with enriched expression patterns within the domains.

The advantages of spaMMCL can be attributed to several key aspects. Firstly, the MML module enhances the model’s integration of different modalities. Secondly, MGL selects specific spatial domains as neighboring regions to the target domain and analyzes regional indicators across various granularities, revealing gene expression dynamics at both micro and macro levels. Lastly, MGL’s analysis relies on spatial domains identified by MML, ensuring the accurate identification of SVGs with spatial expression patterns.

## Supplementary Material

btae607_Supplementary_Data
