# Supplementary material for: A multi-modality and multi-granularity collaborative learning framework for identifying spatial domains and spatially variable genes
Source: Bioinformatics. 2024 Oct 17;40(10):btae607. doi: 10.1093/bioinformatics/btae607 (PMC11513014; doi:10.1093/bioinformatics/btae607)
Supplement: btae607_Supplementary_Data [file btae607_supplementary_data.pdf]

Supplementary Information

A multi-modality and multi-granularity collaborative learning framework for identifying spatial domains and domain-specific spatially variable genes

Xiao Liang[1], Pei Liu[1], Li Xue[1], Baiyun Chen[2], Wei Liu[1], Wanwan Shi[1], Yongwang Wang[1], Xiangtao Chen[1], and Jiawei Luo[1,*]

[1]College of Computer Science and Electronic Engineering, Hunan University, Changsha 410083, China

[2]Department of Computer Science, Tuskegee University

Jiawei Luo: luojiawei@hnu.edu.cn;

1. Supplementary Notes

Supplementary Note 1.

Clustering

Following the clustering process, we adopt the Graphst methodology to implement an optional refinement step for the clustering results. In this step, if more than half of a given spot's neighboring spots are assigned to a different domain, we reassign the spot's label to match the primary label of that neighboring domain. This cluster refinement procedure is applied to all datasets.

The Adjusted Rand Index (ARl) ranges from -1 to 1, with higher values indicating better clustering quality. Given the ground truth class labels e' and the predicted class labels e, the ARl can be calculated using the following formula:

$$ARI(e,e') = \frac{\sum_{p,g}\binom{N_{pg}}{2} - [\sum_p \binom{N_p}{2}\sum_g \binom{N_g}{2}]/\binom{N}{2}}{\frac{1}{2}[\sum_p \binom{N_p}{2} + \sum_g \binom{N_g}{2}] - [\sum_p \binom{N_p}{2}\sum_g \binom{N_g}{2}]/\binom{N}{2}}$$

In this context, N represents the total number of spots, N_g denotes the number of spots within the true cluster g, N_p indicates the number of spots in the predicted cluster p, and N_{pg} refers ta the number of spots that are common to both clusters p and g.

The Normalized Mutual Information (NMI) value ranges from 0 to 1, with higher values indicating greater similarity between the clustering results and the true labels. Let c represent the predicted clustering results and c' denote the ground truth labels. The NMl is defined as follows:

$$NMI(c,c') = \frac{\sum_{i=1}^{k}\sum_{j=1}^{k} n_{ij} \log \frac{n n_{ij}}{n_i n_j}}{\sqrt{\left(\sum_i^k n_i \log \frac{n_i}{n}\right)\left(\sum_j^k n_j \log \frac{n_j}{n}\right)}}$$

where n_{i} and n_{j} denote the number of spots in the true cluster i and the predicted cluster j, separately, and n_{ij} is the number of spots in both clusters i and j.

We selected ARI as our primary metric for two reasons. First, ARI provides a

relatively objective assessment of clustering performance. Second, ARI is a widely accepted measure in cluster analysis. For instance, methods such as SpatialGlue (Long et al., 2024), stDGCC (Zhang et al., 2024) and spaVAE (Tian et al., 2024) use ARI as a principal evaluation indicator. Similarly, our comparison method, such as CCST (Li et al., 2022) and STAGATE (Dong et al., 2022) also utilizes this metric.

References

Long. et al. Deciphering spatial domains from spatial multi-omics with SpatialGlue[J]. Nature Methods, 2024: 1-10.

Zhang. et al. Unraveling spatial domain characterization in spatially resolved transcriptomics with robust graph contrastive clustering[J]. Bioinformatics, 2024, 40(7).

Tian. et al. Dependency-aware deep generative models for multitasking analysis of spatial omics data[J]. Nature Methods, 2024: 1-13.

Li. et al. Cell clustering for spatial transcriptomics data with graph neural networks[J]. Nature Computational Science, 2022, 2(6): 399-408.

Dong. et al. Deciphering spatial domains from spatially resolved transcriptomics with an adaptive graph attention auto-encoder[J]. Nature communications, 2022, 13(1): 1739.

Supplementary Note 2.

Moran's I

Moran's I is a correlation coefficient that quantifies the overall spatial autocorrelation within a dataset. Specifically, for a given gene, it assesses the similarity of one location to its surrounding locations. When neighboring spots exhibit attraction or repulsion, it suggests a lack of independence among them, highlighting the presence of spatial autocorrelation in gene expression patterns. The Moran's I statistic ranges from -1 to 1: values approaching 1 indicate a pronounced spatial pattern, values near 0 suggest random spatial distribution, and values close to -1 reflect a chessboard-like arrangement. To analyze the spatial variability of a specific gene, we calculate Moran's I using the following formula:

$$I = \frac{N}{W} \frac{\sum_i \sum_j [w_{ij}(x_i - \bar{x})(x_j - \bar{x})]}{\sum_i (x_i - \bar{x})^2}$$

In this context, x_i and x_j represent the gene expression levels at spots i and j, respectively, while $\bar{x}$ denotes the mean expression across all spots. N indicates the total number of spots, and w_{ij} refers to the spatial weight between spots i and j,calculated based on their 2D spatial coordinates. The term W represents the sum of all w_{ij} .For each spot, we identify the k nearest neighbors using their spatial coordinates. In our analysis, the Moran's I statistic demonstrates robustness with respect to the choice of k, which we have set at 4. We define $w_{ij} = 1$ if spot j is among the nearest neighbors of spot i, and $w_{ij} = 0$ otherwise.

Supplementary Note 3.

Parameter setting

The spaMMCL was implemented using Python and PyTorch. For the morphological encoder, we utilized ResNet152 with default pretrained weights sourced from torchvision.models. The optimization of our models was performed using the AdamW optimizer with a learning rate of 0.001. During the refinement step, the number of neighboring spots was configured to 50.

In the fine-grained screening strategy, we employed a fold change threshold of 1.5. Fold change measures the magnitude of gene expression variations, with higher values leading to the identification of fewer SVGs. The threshold of 1.5 is a standard and widely adopted value, also utilized by the spaGCN method (Hu et al., 2021), and thus was chosen as our fold change threshold.

In the coarse-grained screening strategy, the threshold for $\mathrm{p}_{\mathrm{adj}}^{\mathrm{C}}$ was set at 0.05. The $\mathrm{p}_{\mathrm{adj}}^{\mathrm{C}}$ represents the significance level of the adjusted p-value. A threshold of 0.05 is a standard and widely accepted value, utilized by various methods including SOMDE (Hao et al., 2021) and BSP (Wang et al., 2023). Therefore, we adopted 0.05 as the threshold for $\mathrm{p}_{\mathrm{adj}}^{\mathrm{C}}$.

The parameter “α” serves as a weighting factor that balances the influence of gene reconstruction loss and other losses. To investigate the influence of parameter “α” on spatial domain identification results, we performed a parameter analysis using the HBC dataset. All other parameters were held constant while “α” was varied from 1 to 19 in increments of 2. As shown in Supplementary Table 1, setting parameter “α” to 10 exhibited optimal performance. Consequently, we selected α = 10 as the optimal parameter setting.

Supplementary Table 1. The parameter experiment results on HBC.

α	1	3	5	7	9	10	11	13	15	17	19
ARI	0.58	0.57	0.57	0.57	0.58	**0.64**	**0.64**	0.53	0.59	0.58	0.59

References

Hu. et al. SpaGCN: Integrating gene expression, spatial location and histology to identify spatial domains and spatially variable genes by graph convolutional network[J]. Nature methods, 2021, 18(11): 1342-1351.

Hao. et al. SOMDE: a scalable method for identifying spatially variable genes with self-organizing map[J]. Bioinformatics, 2021, 37(23): 4392-4398.

Wang. et al. Dimension-agnostic and granularity-based spatially variable gene identification using BSP[J]. Nature Communications, 2023, 14(1): 7367.

2. Supplementary Figures

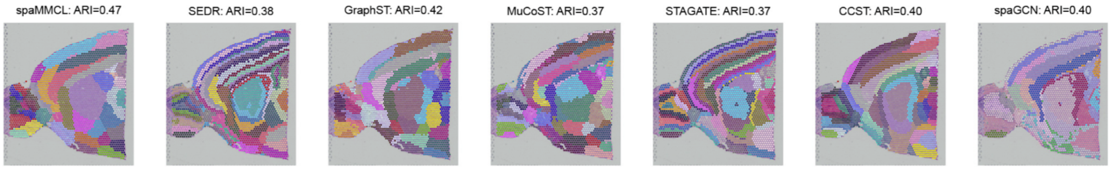

Supplementary Figure 1. Domain identification by all methods (Mouse Brain)

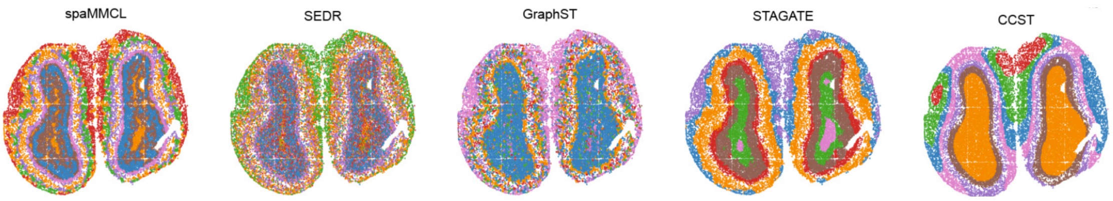

Supplementary Figure 2. Domain identification by all methods (Mouse olfactory bulb)

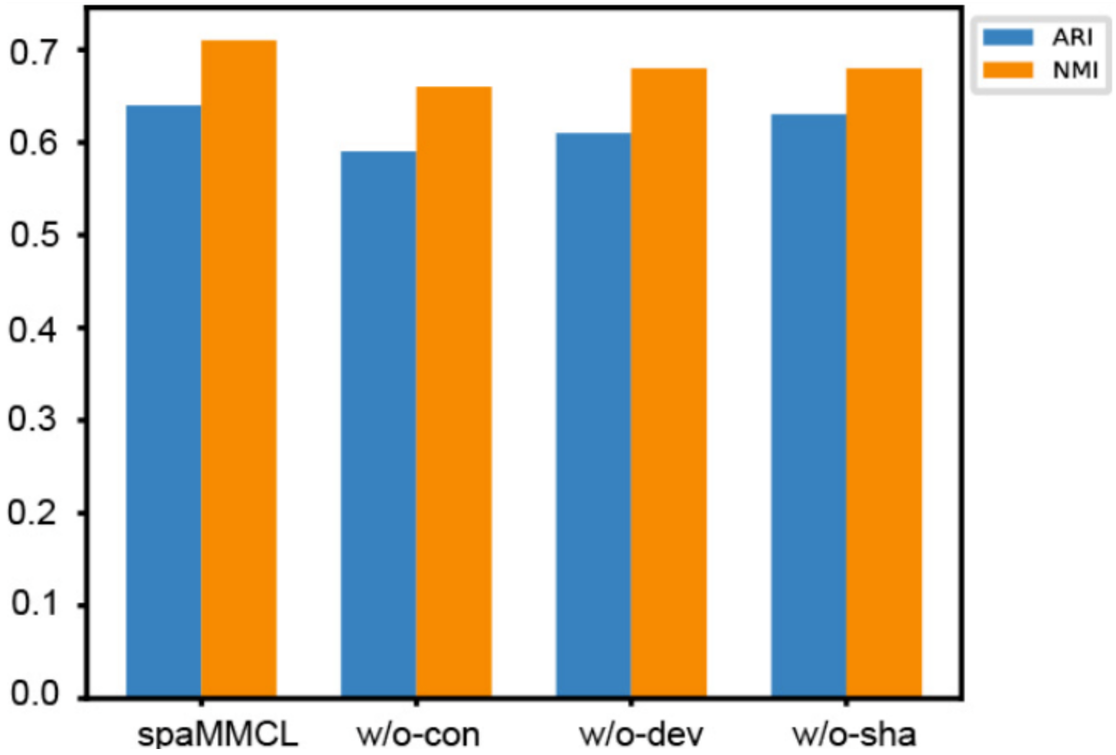

Supplementary Figure 3. The histogram of ARI and NMI for spaMMCL and its variants.

3. Supplementary Datasets

Supplementary Table 2. Summary of the datasets

**Data**	**Section**	**clusters**	**Spots**	**Genes**
Human dorsolateral prefrontal cortex	151507	7	4226	33538
	151508	7	4384	
	151509	7	4789	
	151510	7	4634	

	151669	5	3661	
	151670	5	3498	
	151671	5	4110	
	151672	5	4015	
	151673	7	3639	
	151674	7	3673	
	151675	7	3592	
	151676	7	3460	
Human breast cancer	/	20	3798	36601
Mouse Brain	Anterior	52	2695	32285
	Posterior	/	3355	
Mouse olfactory bulb	/	/	19893	27106
